# Anthracyclines, proteasome activity and multi-drug-resistance

**DOI:** 10.1186/1471-2407-5-114

**Published:** 2005-09-13

**Authors:** Mirela R Fekete, William H McBride, Frank Pajonk

**Affiliations:** 1 Department of Neurology, Bürgerhospital, Tunzhofer Str. 14-16, 70191 Stuttgart, Germany; 2 Department of Radiation Oncology, Roy E. Coats Labs., David Geffen School of Medicine at UCLA, 10833 Le Conte Avenue, Los Angeles, CA90095-1714, USA

## Abstract

**Background:**

P-glycoprotein is responsible for the ATP-dependent export of certain structurally unrelated compounds including many chemotherapeutic drugs. Amplification of P-glycoprotein activity can result in multi-drug resistance and is a common cause of chemotherapy treatment failure. Therefore, there is an ongoing search for inhibitors of P-glycoprotein. Observations that cyclosporin A, and certain other substances, inhibit both the proteasome and P-glycoprotein led us to investigate whether anthracyclines, well known substrates of P-gp, also inhibit the function of the proteasome.

**Methods:**

Proteasome function was measured in cell lysates from ECV304 cells incubated with different doses of verapamil, doxorubicin, daunorubicin, idarubicin, epirubicin, topotecan, mitomycin C, and gemcitabine using a fluorogenic peptide assay. Proteasome function in living cells was monitored using ECV304 cells stably transfected with the gene for an ubiquitin/green fluorescent protein fusion protein. The ability of the proteasome inhibitor MG-132 to affect P-glycoprotein function was monitored by fluorescence due to accumulation of daunorubicin in P-glycoprotein overexpressing KB 8-5 cells.

**Results:**

Verapamil, daunorubicin, doxorubicin, idarubicin, and epirubicin inhibited 26S chymotrypsin-like function in ECV304 extracts in a dose-dependent fashion. With the exception of daunorubicin, 20S proteasome function was also suppressed. The proteasome inhibitor MG-132 caused a dose-dependent accumulation of daunorubicin in KB 8-5 cells that overexpress P-glycoprotein, suggesting that it blocked P-glycoprotein function.

**Conclusion:**

Our data indicate that anthracyclines inhibit the 26S proteasome as well as P-glycoprotein. Use of inhibitors of either pathway in cancer therapy should take this into consideration and perhaps use it to advantage, for example during chemosensitization by proteasome inhibitors.

## Background

Multi-drug-resistance (MDR) is a common reason for chemotherapy treatment failure in breast cancer, leukemia, and non-Hodgkin lymphoma patients. MDR can often be attributed to over-expression of the mdr1 gene that codes for an ATP-dependent, transmembrane P-glycoprotein (P-gp) efflux pump pathway, which rapidly exports man structurally un-related drugs from the cell, including anthracyclines [1,2].

Numerous pre-clinical and clinical studies using P-gp modulating compounds like verapamil, cyclosporin A, reserpine, staurosporine, propafenone, phenoxazine, chloroquine, phenothiazine and their derivates have been undertaken to overcome MDR and several substances have been identified that are effective *in vitro *(reviewed in [3]). However, to revert MDR *in vivo*, most MDR-modulating drugs require serum concentrations that have unacceptable toxicity and therefore they are currently not used in standard chemotherapy regimens. The development of better, less toxic inhibitors might be aided by insights into the specificity of these inhibitors for other molecules and the spectrum of molecules bound by P-glycoprotein.

Two of the most commonly used MDR-modulating substances are verapamil and cyclosporin A (CsA), or their derivates. Interestingly, CsA has recently been identified as an inhibitor of the 26S proteasome [4]. The 26S proteasome is a highly conserved multicatalytic protease responsible for ATP- and ubiquitin-dependent degradation of all short-lived and 70–90% of all long lived proteins including cyclin A, B and E, p21 and p27, p53, cJun, cFos, and IκB. As such, the 26S proteasome controls cell cycle, signal transduction pathways, apoptosis and major functions of the immune system. Indeed some of the immunosuppressive properties of CsA, such as decreases in the expression of MHC-I molecules on the surface of target cells [5] and apoptotic death of lymphocytes through inhibition of the transcription factor NF-κB [6], may be due to its inhibitory effect on proteasome function. Vinblastine, a known P-gp substrate has also been shown to inhibit proteasome activity [7]. And, remarkably, the HIV protease inhibitor ritonavir was identified as an inhibitor of P-gp [8] and the proteasome [9]. Since CsA and ritonavir have been shown to inhibit both proteasome and P-gp activities, we questioned whether there was cross specificity between P-gp and proteasome activities. Cross specificity might explain effects of P-gp inhibitors on multiple cellular parameters that seem extrinsic to a pumping function of P-gp. Insights into substrate cross specificity of P-gp could offer a basis for the development of more selective P-gp inhibitors. They could also indicate reasons for the toxicity of these inhibitors, and why they affect cellular functions other than those related to P-gp.

Using an *in vitro *model, we show that anthracyclines and verapamil both inhibit proteasome function. Additionally, we demonstrate that the proteasome inhibitor MG-132 inhibits P-gp function, thereby increasing the uptake of doxorubicin in the cytoplasm and the nucleus.

## Methods

### Cell culture

KB 8.5 human epitheloid carcinoma cells that overexpress P-gp were a generous gift from Dr. Peter Hafkemeyer (University Clinic Freiburg, Germany). Every 21 days P-gp-positive KB 8.5 cells were selected by addition of colchicine (10 ng/ml, Sigma). 24 hours before drug treatment cells were plated into 6-well plates (Costar) at a density of 10^6 ^cells/well.

EVC 304 human bladder carcinoma cells and PC-3 prostate cancer cells were obtained from the German Microorganism and Tissue Culture Collection (German collection of microorganism and cell cultures, DSMZ, Braunschweig). Cells were grown in 75 cm^2 ^flasks (Falcon) at 37°C in a humidified atmosphere at 5 % CO_2 _in DMEM medium (Sigma) supplemented with 10 % heat inactivated FCS (Sigma) and 1 % penicillin/streptomycin (Gibco BRL).

### Drug treatment

Stock solutions of all cytotoxic drugs were obtained from the hospital pharmacy of the University Clinic Freiburg. MG-132 (Calbiochem) was dissolved at 10 mM in DMSO and stored as small aliquots (10–30 μl) at -20°C. In drug accumulation assays doxorubincin (10 μM), daunorubicin (2–16 μM) or MG-132 (0.5–50 μM, 0.5% DMSO) were added to cells at the indicated times. Control cells were subjected to DMSO treatment alone (0.5 %).

### Proteasome function assays

20S and 26S proteasome function was measured as described previously (20). Briefly, cells were washed with PBS, then with buffer I (50 mM Tris, pH 7.4, 2 mM DTT, 5 mM MgCl_2_, 2 mM ATP), and pelleted by centrifugation. Glass beads and homogenization buffer (50 mM Tris, pH 7.4, 1 mM DTT, 5 mM MgCl_2_, 2 mM ATP, 250 mM sucrose) were added and vortexed for 1 minute. Beads and cell debris were removed by centrifugation at 1,000 × g for 5 minutes and 10,000 × g for 20 minutes. Protein concentration was determined by the BCA protocol (Pierce). One hundred μg protein of each sample was diluted with buffer I to a final volume of 1000 μl and the fluorogenic proteasome substrate SucLLVY-7-amido-4-methylcoumarin (chymotrypsin-like, Sigma) was added in a final concentration of 80 μM in 1% DMSO. To access 20S function, buffer I was replaced by an ATP-free buffer containing SDS (20 mM HEPES, pH 7.8; 0.5 mM EDTA, 0.03% SDS) [10]. Cleavage activity was monitored continuously by detection of free 7-amido-4-methylcoumarin using a fluorescence plate reader (Gemini, Molecular Devices) at 380/460 nm and 37°C. As controls for drug studies, 7-amido-4-methylcoumarin (AMC, 2 μM) was incubated with drugs in buffer I without cell extracts and measurements of proteasome function were corrected when necessary.

### Drug accumulation assay

Total cellular daunorubicin content and accumulation of doxorubicin in the cytoplasm and nucleus were determined as described elsewhere [11] with some minor modifications. Growth medium on cells was replaced by PBS for 40 minutes at 37°C. This was replaced by fresh PBS containing daunorubicin or doxorubicin and MG-132 or anthracyclines alone. In some experiments, cells were washed with PBS after daunorubicin treatment and incubated in PBS containing MG-132 for an additional 40 minutes at 37°C. After drug treatment, the cells were washed twice with PBS, re-suspended in either 4 ml lysis buffer (0.3 M sucrose, 0.05 mM EGTA pH 8.0, 60 mM KCl, 15 mM NaCl, 15 mM HEPES pH 7.5, 150 μM spermine, 50 μM spermidine) containing 20 μl triton X-100 for nuclear isolation or 400 μl of 50% ethanol in 1 M HCl (v/v) for whole cell lysis. For the latter, cells were vortexed and diluted with water to a final volume of 1.4 ml. The cells in lysis buffer were mixed and left on ice for 15 minutes before centrifuging. The nuclei (pellet) were then vortexed with 400 μl HCl/isopropanol. Fluorescence derived from daunorubicin or doxorubicin was measured in quadruplicates of 200 μl using a fluorescence plate reader (Gemini, Molecular Devices) at 480/575 nm.

### Transfection

ECV304 cells were plated at a density of 250.000 cells/well into six-well plates twelve hours before transfection. Cells were transfected with 5 μg of a plasmid (pEGFP-N1, Clontech) coding for an ubiquitin (Ub)-R-GFP fusion protein under control of a CMV promoter [12] (a kind gift from Dr. M. Masucci, Karolinska Institute, Sweden) using the Superfect transfection kit (Qiagen) and following the manufacturer's instructions. Transfected cells were maintained in DMEM (10 % FSC, 1 % penicillin/streptomycin) supplemented with 500 μg/ml G418 (Sigma) and clones were obtained. Expression of Ub-R-GFP was analyzed by flow cytometry (FL1-H, *FACSCalibur*, Becton Dickinson) using *CellQuest *Software before and after treatment with the proteasome inhibitor MG-132 (50 μM) for 10 hours at 37°C. Clone #10 (ECV304/10), which showed low background and high MG-132-induced expression of Ub-R-GFP, was used for inhibition experiments.

### Statistics

Experimental data are presented as mean ± standard error of the mean from at least three independent experiments. A p-value <0.05 in a two-sided student's t-test was considered as 'statistically significant'.

## Results

### Verapamil is an inhibitor of 20S and 26S proteasome function

**Figure 1:**
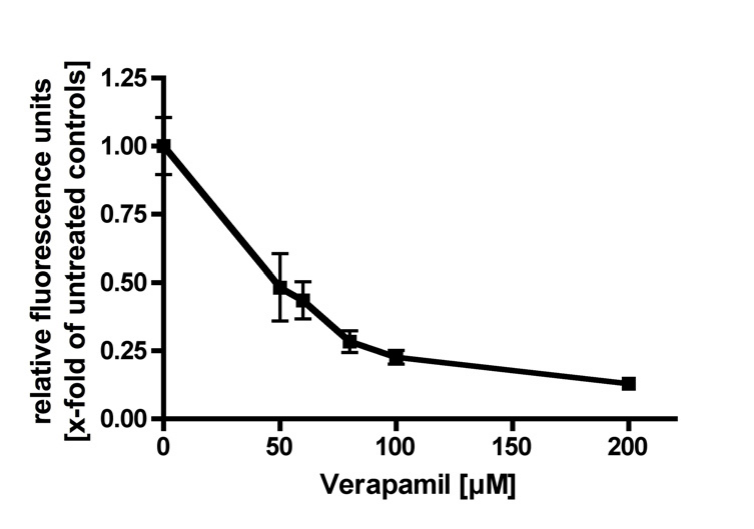
*Verapamil is an inhibitor of 26S proteasome function. *Incubation of crude extracts of ECV304 cells containing proteasomes with different doses of verapamil (50, 60, 80, 100, 200 μM) inhibited proteolysis of the chymotrypsin-like substrate SucLLVY-AMC in a dose-dependent manner, indicating inhibition of 26S proteasome function.

In order to test the hypothesis that the P-gp inhibitor verapamil inhibits proteasome function, proteasome extracts of ECV304 and PC-3 cells were incubated with different concentrations of the drug (0, 50, 100 and 200 μM) and immediately tested for their chymotrypsin-like activity against the fluorogenic substrate SucLLVY-7-amido-4-methylcoumarin. There was a dose-dependent inhibition of MG-132-sensitive 26S (Fig. 1A) and 20S (data not shown) proteasome function, consistent with a direct inhibitory effect of verapamil on the proteasome.

### Anthracyclines inhibit 20S and 26S proteasome function in a dose-dependent manner

Since verapamil, vinblastine, and CsA have been found to inhibit 20S and 26S proteasome function and vinblastine and CsA serve as substrates of P-gp [3], we asked if anthracyclines in general have an inhibitory effect on this protease. When crude extracts of ECV304 cells were incubated with different doses (0 – 100 μM) of the anthracyclines doxorubicin, daunorubicin, idarubicin and epirubicin we observed dose-dependent inhibition of 26S proteasome function with IC_50 _values of 65.5 μM for doxorubicin, 13.7 μM for daunorubicin, 38.6 μM for idarubicin and 29.2 μM for epirubicin (Table 1). Topotecan, mitomycin C, and gemcitabine had no measurable effect on 26S proteasome function (data not shown). 20S proteasome function was inhibited by doxorubicin (IC_50 _5.8 μM), idarubicin (IC_50 _92 μM), epirubicin (IC_50 _12.5 μM) but not by daunorubicin (Table 2).

**Figure 2:**
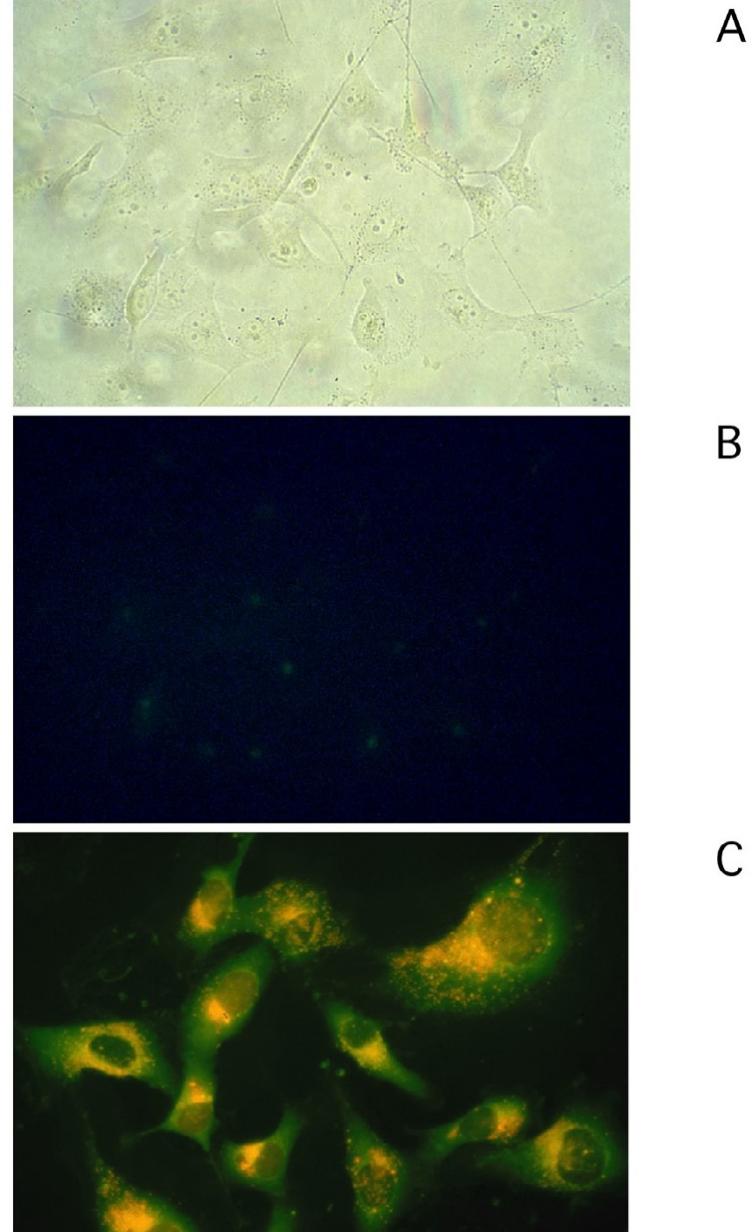
*Anthracyclines are inhibitors of proteasome function. *Incubation of ECV304 cells stably transfected with an Ub-GFP fusion protein with daunorubicin (100 μM, 16 h), caused accumulation of GFP throughout the cytoplasm (lower picture), indicating proteasome inhibition in living cells while untreated controls cells showed only little accumulation of GFP **(A/B)**. Daunorubicin accumulated in the perinuclear region **(C)**.

In order to demonstrate if this inhibition could be observed in living cells, we incubated ECV304/10 cells, stably transfected with an expression plasmid for an Ub-GFP fusion protein with doxorubicin (100 μM) for 12 hours. When analyzed by fluorescence microscopy, the cells showed perinuclear accumulation of doxorubicin while GFP accumulated throughout the cytoplasm, indicating inhibition of proteasome function (Fig. 2).

### MG-132 treatment reverts multi-drug-resistance in P-gp expressing KB 8-5 cells

**Figure 3:**
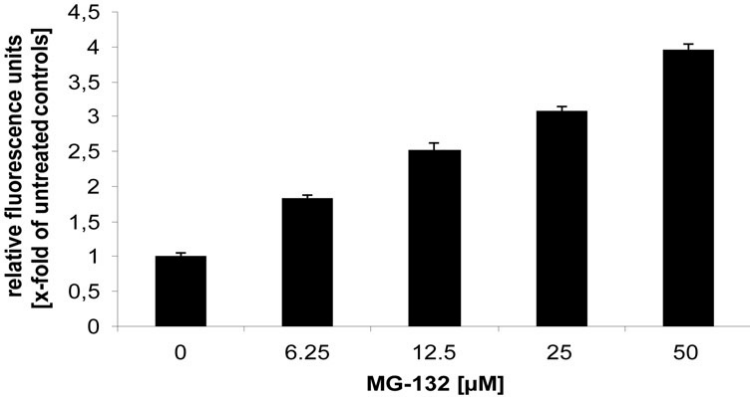
*MG-132 treatment of KB 8-5 causes intracellular accumulation of anthracyclines. *Incubation of KB 8-5 cells, which overexpress P-gp, with increasing doses of MG-132 (0, 6.25, 12.5, 25, 50 μM) caused a dose-dependent accumulation of daunorubicin, as measured by fluorescence, indicating inhibition of P-gp function by this proteasome inhibitor.

**Figure 4:**
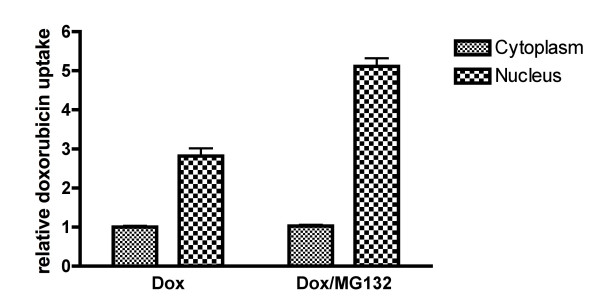
Accumulation of doxorubicin in the presence or absence of MG-132 (25 μM) in the cytoplasm and the nuclear fraction of ECV304 cells.

The human epitheloid carcinoma cell line KB 8-5 is a well-characterized tumor cell line that over-expresses mdr-1 with associated MDR. Preliminary experiments showed that treatment of KB 8.5 cells with the reversible proteasome inhibitor MG-132 (3.125 to 50 μM) induced apoptosis within 24 hours. This is in accord with numerous studies reporting induction of apoptosis in cancer cells by proteasome inhibitors [13], and indicated that MG-132 enters KB 8-5 cells and that they are not abnormally resistant to its effects based on enhanced P-gp function. After 45 minutes of incubation with MG-132 (50 μM), no morphological signs of toxicity were observed. KB 8-5 cells treated with different doses of MG-132 and daunorubicin (10 μM) for 45 minutes showed increased, dose-dependent accumulation of daunorubicin in the cytoplasm (e.g. a 4-fold increase at 50 μM MG-132, Fig. 3) indicating that MG-132 could block P-gp function. This was further supported by the observation that incubation of ECV304 cell with MG-132 (25 μM) caused an increased uptake of doxorubicin in the cytoplasm and in the nuclear fraction of the cells (Fig. 4).

## Discussion

The observations that CsA [4] and vinblastine [7] have inhibitory effects on the cleavage activity of the 26S proteasome led us investigate the effects of anthracycline anticancer agents and verapamil on the activity of this protease. Verapamil caused a concentration-dependent inhibition of 20S and 26S function. Additionally, we found a concentration-dependent inhibition of 26S proteasome function for all four anthracyclines tested. Comparable results showing doxorubicin to be a non-competitive inhibitor of the proteasome have been reported previously [14]. With the exception of daunorubicin, anthracyclines also inhibited 20S chymotryptic function in a dose-dependent manner. It is known that doxorubicin is co-transported into the nucleus along with proteasomes [15,16] but our observation of a general direct inhibitory effect of anthracycline anticancer agents on the proteasome sheds a totally new light on the actions of these drugs.

The inhibitory effects of the reversible inhibitor of the proteasome, MG-132, on P-glycoprotein function, supports the view that P-glycoprotein and the proteasome can both be targeted by this new class of chemotherapeutic drugs. This was further supported by the observation that verapamil, another established inhibitor of P-gp, inhibited the chymotryptic 20S and 26S function of the proteasome. The fact that both P-glycoprotein and proteasome activities can both be regulated by pro-inflammatory cytokines and oxidative stress suggests [17-21] that studies on co-ordinate regulation of these activities might be illuminating.

These findings lead to interesting possibilities with respect to the possible use of proteasome inhibitors, which are just entering their first clinical trials [22,23], in combination therapy, as well as to the mechanism of action and toxicity of P-gp inhibitors. Using an *in vitro *system, we showed that the proteasome inhibitor MG-132 caused intracellular accumulation of anthracyclines, indicating inhibition of P-gp function. Proteasome inhibitors may interfere with drug-resistance at additional levels as P-gp and also topisomerase II are degraded in a proteasome-dependent manner and degradation is blocked by proteasome inhibitors [24,25]. However, given the long half-life of P-gp of 14–24 hours [26], the effects observed in our study after short-time incubation of the cells with MG-132 are probably not caused by an increased degradation of P-gp. The extent of the increase of anthracyclin accumulation in mdr1-overexpressing KB-8.5 treated with 25 μM concentrations of MG-132 cells in our study was comparable to the effect of verapamil at 50 μM [27]. Future studies have to clarify if similar effects can be obtained using clinically used proteasome inhibitors at concentrations typically reached in the serum of patients.

Tumor cells in general exhibit altered patterns of expression of proteasome subunits and their distribution between cytoplasm and nucleus often differs from normal cells [28-30]. This may explain why specific proteasome inhibitors like PS-341 are usually clinically well tolerated. Inhibition of proteasome function induces apoptosis of tumor cells [31-34] and sensitizes the surviving tumor cells to the actions of both chemotherapy [35] and radiation therapy [36,37]. Therefore, proteasome inhibitors might overcome P-gp-related MDR, with accompanying chemo- and radiosensitizing effects. Also, since tumor microvasculature expresses high levels of mdr-1 [38,39], the possibility exists that the neovasculature is a target for these drugs in vivo. On the other hand, direct inhibition of proteasome function might be an additional major mechanism of action for anthracyclines. Such inhibition could contribute to their ability to enhance the efficacy of other chemotherapeutic drugs, independent of their ability to reverse MDR.

## Competing interests

The author(s) declare that they have no competing interests.

## Authors' contributions

MF carried out the molecular studies and helped to draft the manuscript. WMB participated in the design of the study and helped to draft the manuscript. FP designed the study and drafted the manuscript. All authors read and approved the final manuscript.

## Pre-publication history

The pre-publication history for this paper can be accessed here:



## Figures and Tables

**Table 1:** Chymotryptic 26S Proteasome Activity in Lysates from ECV 304 Cells

μM	Doxorubincin	Daunorubicin	Epirubicin	Idarubicin
0	1	1	1	1
6.25	0.92 ± 0.03*	0.85 ± 0.05*	0.79 ± 0.1 n.s.	0.97 ± 0.03 n.s.
12.5	0.84 ± 0.05*	0.53 ± 0.11*	0.57 ± 0.31 n.s.	0.96 ± 0.02 n.s.
25	0.72 ± 0.04**	0.22 ± 0.09**	0.59 ± 0.14*	0.79 ± 0.12 n.s.
50	0.59 ± 0.1*	0.12 ± 0.06**	0.36 ± 0.2*	0.28 ± 0.09 **
100	0.36 ± 0.03***	0.11 ± 0.06**	0.27 ± 0.17*	0.1 ± 0.06 **

n.s. not significant, *p < 0.05, **p < 0.01, ***p < 0.001 (two-sided student's t-test)

**Table 2:** Chymotryptic 20S Proteasome Activity in Lysates from ECV 304 Cells

μM	Doxorubincin	Daunorubicin	Epirubicin	Idarubicin
0	1	1	1	1
6.25	0.49 ± 0.1*	0.62 ± 0.19 n.s.	0.71 ± 0.21 n.s.	0.67 ± 0.07*
12.5	0.28 ± 0.09**	0.64 ± 0.07 n.s.	0.46 ± 0.2 n.s.	0.67 ± 0.08*
25	0.28 ± 0.13*	0.81 ± 0.05 n.s.	0.37 ± 0.08**	0.69 ± 0.05**
50	0.24 ± 0.06**	0.95 ± 0.04 n.s.	0.33 ± 0.07**	0.67 ± 0.05 **
100	0.25 ± 0.07**	1.05 ± 0.1 n.s.	0.35 ± 0.07**	0.49 ± 0.11*

n.s. not significant, *p < 0.05, **p < 0.01, ***p < 0.001 (two-sided student's t-test)
